# The Correlation Between Vitamin D Deficiency and Chronic Rhinosinusitis: A Systematic Review

**DOI:** 10.7759/cureus.55955

**Published:** 2024-03-11

**Authors:** Ghaida Nawi A Alharthi, Ali Alzarei

**Affiliations:** 1 Department of Otolaryngology, Head and Neck Surgery, King Khalid University, Abha, SAU; 2 Otolaryngology - Head and Neck Surgery, Aseer Central Hospital, Abha, SAU

**Keywords:** chronic rhinosinusitis, crswnp, deficiency, correlation, vitamin d, crs

## Abstract

Chronic rhinosinusitis (CRS) is marked by inflammation of the paranasal sinuses and mucosal lining of the nose. CRS can be classified as CRS with nasal polyps or CRS without polyps. In recent years, there has been increased attention on the immunological role of vitamin D in treating CRS, coupled with the observation of vitamin D deficiency among CRS patients. Vitamin D is acknowledged for its immunomodulatory properties, especially in allergic conditions. Recent studies suggest that individuals with CRS, especially those with CRS with nasal polyps, frequently demonstrate lower than normal levels of vitamin D. We conducted searches across various databases, including PubMed, Web of Science, ScienceDirect, and the Cochrane Library, both electronically and manually, to identify pertinent studies. Studies from 2003 to 2023 were included. Deficiency of vitamin D was considered with levels of vitamin D less than 30 ng/mL, and only original studies were included. Studies including patients taking vitamin D supplementation previously and patients with chronic kidney and liver diseases were excluded. We included nine studies that recruited 1,042 patients in total. More than half of the studies reported the presence of nasal polyps in CRS, and participants from four studies had CRS without nasal polyps. All of the included studies reported a negative correlation between levels of vitamin D and CRS. The majority of the studies depicted quite low levels of vitamin D among CRS patients. The degree of CRS severity as measured by endoscopic and radiological scores is moderately inversely correlated with vitamin D intake. Therefore, it is recommended that the relationship between various serum vitamin D concentrations and the severity of CRS be investigated in detail, along with an understanding of the cellular effects of vitamin D levels on the nasal mucosa.

## Introduction and background

Chronic rhinosinusitis (CRS) is an upper respiratory tract ailment marked by widespread inflammation of the mucosal lining of the nose and paranasal sinuses and is one of the most common chronic diseases in the world. It is a serious health issue whose prevalence and incidence are increasing. In many circumstances, CRS is still challenging to manage and places a significant economic cost on society. CRS not only imposes a significant economic burden but also profoundly affects the quality of life for affected individuals. The impact extends beyond financial costs, encompassing physical discomfort, emotional strain, and limitations in daily activities. It can lead to decreased productivity, social withdrawal, and diminished overall well-being. CRS is a complex disease with a proposed etiology, including anatomical factors, infectious causes, immunological complications, biofilms, and genetic causes. Chronic inflammation of the sinonasal mucosa is the inevitable outcome of all these pathophysiological causes [1]. Epidemiological studies from across the globe indicate that considering either symptoms or objective findings alone has resulted in the prevalence estimates for CRS being almost 10%; however, following the guideline-based diagnostic criteria which requires both symptoms and objective findings exhibits that the actual prevalence of CRS is consistently under 5%. Furthermore, it is noted that about one-third of individuals diagnosed with CRS also have nasal polyps [2].

CRS is categorized into the following two types: CRS with nasal polyps and CRS without polyps. The main symptoms differentiating these two forms are facial pain and a loss of smell. Both symptoms are indicative of a significant disease impact and share a range of clinical presentations [3]. The chronic inflammatory and tissue-deforming processes that characterize CRS are thought to be influenced by changes in mucociliary clearance, anomalies in the sinonasal epithelial cell barrier, and tissue remodeling. The host’s innate and adaptive immune systems are also highly active and may contribute significantly to the disease [4]. The four cardinal symptoms of blockage, drainage, loss of smell, and facial pain or pressure are used to diagnose rhinosinusitis in both acute and chronic cases, with CRS being characterized by a minimum three-month symptom duration [5].

The pathophysiology of vitamin D is a remarkable advancement in the field of upper respiratory tract diseases. There is currently extensive evidence from research that suggests vitamin D3 functions locally inside the respiratory epithelium as an immunomodulator of innate and adaptive immunity [6]. Globally, vitamin D3 insufficiency is a cause for concern. Sunscreen use, time spent outdoors, obesity rates, and population growth in northern latitudes have been identified as significant factors. Vitamin D3 appears to play a role in the control of the immune system in addition to its well-described role in calcium homeostasis by triggering innate antibacterial responses while typically having a tolerogenic effect on adaptive immunity. In recent times, its function in the etiology of CRS with nasal polyps is becoming clearer. Sinonasal epithelial cells reportedly express 1-hydroxylase and have the ability to locally generate 1,25-vitamin D3. In patients with CRS with nasal polyps, the deficiency/lack of this molecule probably results in diminished antibacterial responses, increased production of inflammatory cytokines, and enhanced fibroblast proliferation [7]. In this study, we aim to collect all potential evidence from relevant studies that reported the correlation between vitamin D deficiency and CRS.

## Review

Methodology

Definition of Outcomes and Inclusion Criteria

Our objective was to assess and evaluate the correlation between a deficiency of vitamin D and CRS. Consequently, we included original investigations that recruited patients with CRS at baseline with confirmed and reported vitamin D deficiency. There is no universally accepted normal or optimal value for vitamin D, yet its beneficial effects are most noticeable when its levels are approximately 30 ng/mL. Hence, a deficiency of vitamin D is considered at vitamin D levels of less than 30 ng/mL. Our search encompassed studies published between 2003 and 2023. Studies reporting patients taking vitamin D supplementation previously and patients with chronic kidney and liver diseases were excluded. Moreover, case reports with limited sample sizes and no descriptive statistics were also excluded from this review. Other exclusion criteria were nonhuman or laboratory studies, nonoriginal investigations or incomplete studies, abstract-only articles, protocols, theses, and articles that were not published in English or with no available English information.

Search Strategy

After obtaining our desired outcomes, we performed a brief manual screening of potentially included studies to identify relevant keywords for the most appropriate search term. Our search terms included (‘Chronic rhino sinusitis’ OR CRSwNP OR ‘nasal polyps’ OR sinusitis) AND (‘Sinonasal outcome test-22’ OR ‘Radiographic signs’ OR ‘Lund-Mackay Scale)’ AND (correlation OR association OR relationship) AND (Vitamin D OR ‘Vitamin D3’ OR Calcitriol OR Ergocalciferol OR Cholecalciferol) AND (deficiency OR insufficiency) AND (‘Vitamin D deficiency’) databases, including PubMed, Google Scholar, Web of Science and Science Direct. We limited our search to the titles and abstracts of search outcomes to capture all pertinent studies. Subsequently, these results were organized into an Endnote library, where we eliminated duplicate entries from various databases. Moreover, we conducted a manual review of the reference lists from the studies and relevant reviews included, along with the related articles sections on PubMed, to uncover any studies that our electronic search might have overlooked. Throughout this systematic review, we adhered to the guidelines set forth by the Preferred Reporting Items for Systematic Reviews and Meta-Analyses (PRISMA) [8].

Screening and Extraction

To maintain the integrity and quality of our review, we employed a dual screening approach, which encompassed an examination of both titles and abstracts, as well as the full texts of papers. This screening was performed independently by two reviewers in a blind process, with a senior member overseeing the procedure and mediating any disagreements that arose between reviewers. An extraction form was developed to systematically capture information aligned with our research goals, including baseline characteristics of studies, publication details, abstracts, decisions regarding article inclusion or exclusion, and justifications for those exclusions. We also distinguished whether each article was a clinical trial. Our selection process was rigorous, ensuring the inclusion of all articles that fulfilled our specified criteria.

Quality Assessment

Information was collected from the selected studies to evaluate their potential risk of bias. For the appraisal of observational studies, we utilized the adapted Newcastle-Ottawa scale (NOS), which evaluates the following four areas: methodological quality, selection of participants, measurement, and outcome/exposure reporting [9]. The studies received scores from 0 to 10, reflecting the extent of bias present. In the case of the two randomized control trials reviewed, the JADAD scale was applied to determine their quality. This scale examines the risk of bias across the following six aspects: randomization process, blinding effectiveness, criteria for participant inclusion and exclusion, tracking of withdrawals and dropouts, statistical analysis rigor, and the clarity of outcome data reporting [10]. The scoring for articles ranged from 0 (lowest possible quality) to 8 (highest possible quality), with scores between 4 and 8 denoting high-quality studies, ranging from good to excellent in terms of methodological rigor.

Results

Search Results

**Figure 1:**
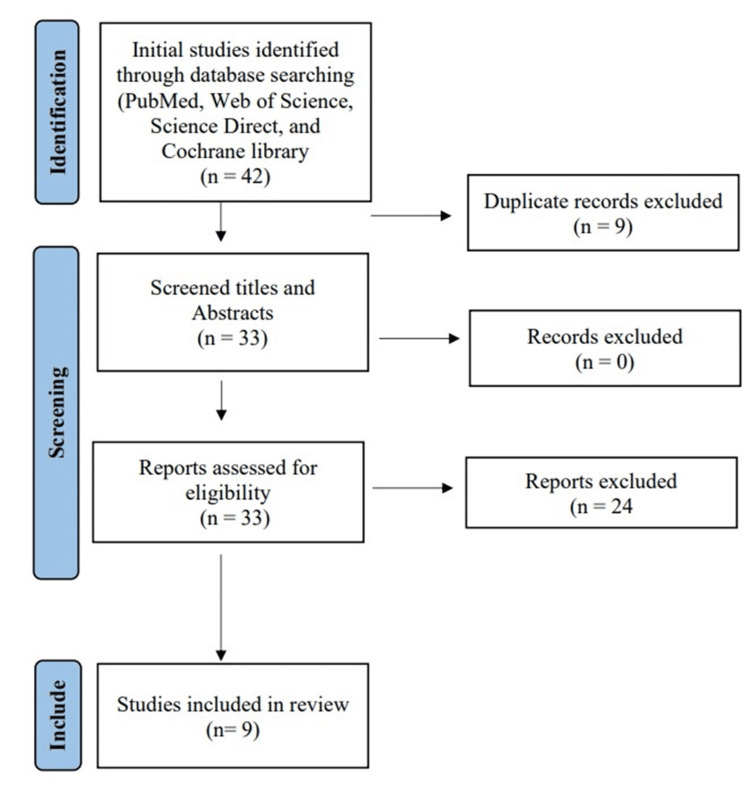
Preferred Reporting Items for Systematic Reviews and Meta-Analyses flow diagram.

Using the search strategies described above, we identified a total of 42 articles, which were then reduced to 33 after removing duplicates. After screening titles and abstracts, only 33 articles were considered eligible for the next steps. Full-text screening narrowed down the number of articles to nine that matched our inclusion and exclusion criteria. Figure 1 shows the detailed search strategy and screening process.

Results of Quality Assessment

The evaluation of study quality indicated that all nonrandomized observational studies were of satisfactory quality, with none achieving excellent or poor ratings. The application of the JADAD scale to the two trials included in the review also indicated a generally low risk of bias. Comprehensive outcomes from the quality assessments, utilizing both the NOS and the adapted JADAD scale, are detailed in Table 1 and Table 2, respectively.

**Table 1:** Newcastle-Ottawa Scale scores of each included non-randomized observational study. scores ranging from 0 to 10 with 0 being the lowest, and 10 the highest

Study	Selection	Comparability	Outcome	Total quality score
Bavi et al. [11]	3	0	3	6
Chandrakar et al. [12]	3	0	3	6
Zand et al. [13]	3	0	3	6
Wang et al. [14]	3	0	3	6
Kalińczak-Górna et al. [15]	3	0	3	6
Tomaszewska et al. [16]	4	0	2	6
Sansoni et al. [17]	4	0	2	6

**Table 2:** Modified Jadad Scale scores of the included randomized controlled trials 1: yes, 2: no

Authors	Was the research described as randomized?	Was the approach of randomization appropriate?	Was the research described as blinding?	What was the approach of blinding?	Was there a presentation n of withdrawals and dropouts?	Was there a presentation of the inclusion/exclusion criteria?	Was the approach used to assess adverse effects described?	Was the approach of statistical analysis described?	Total
Hashemian et al. [18]	1	1	1	1	1	1	1	1	8
Ghazavi et al. [19]	1	1	0	0	1	1	0	1	5

Characteristics of the Included Studies

We included nine studies that recruited 1,042 patients and were published between 2003 and 2023 [11-19]. The total population comprised 536 males and 466 females, with only one study not reporting gender distribution/segregation. The majority of the studies were observational and cross-sectional, with only three randomized control trials. Regarding the geographical distribution of the included studies, the majority were from Iran, followed by two studies from the United States, and one study each from China, India, and Poland. All baseline characteristics of these studies are presented in Table 3. The variations in the sample size of the included articles are likely due to the objective of the specific study and the design.

**Table 3:** Baseline characteristics of the included studies CRSsNP: chronic rhinosinusitis without nasal polyps; CRSwNP: chronic rhinosinusitis with nasal polyps

Author	country	Year	Study	Total participants (n)	Mean age (mean ± SD)	Gender (male/female) (n)
Bavi et al. [11]	Iran	2019	Cross-sectional study	338, CRSwNP: 166, control: 172	CRSwNP: 41.04 ± 13.0, control: 41.00 ± 14.8	191/147
Chandrakar et al. [12]	India	2020	Cross-sectional study	160, case: 80, control: 80	Case: 38.30 ± 10.92, control: 36.40 ± 10.61	95/65
Zand et al. [13]	Iran	2020	Prospective cross-sectional study	93	37.7 ± 13.6	55/38
Wang et al. [14]	China	2019	Retrospective analysis	88, CRSwNP: 42, CRSsNP: 25, control: 21	CRSwNP: 46.50 ± 14.68, CRSsNP: 41.56 ± 15.54, control: 39.61 ± 12.67	46/42
Kalińczak-Górna et al. [15]	Poland	2021	Comparative study	40	NR	NR
Tomaszewska et al. [16]	United States	2019	Prospective study	166, CRSsNP: 52, CRSwNP: 55, control group: 59	CRSsNP: 42.5, CRSwNP: 50.1, control: 36.3	63/103
Sansoni et al. [17]	United States	2015	Nonrandomized clinical trial	57, controls: 12, CRSsNP: 31, CRSwNP: 14	48.7 ± 18.9	26/31
Hashemian et al. [18]	Iran	2020	Randomized controlled trial	40, vitamin D: 20, placebo: 20	Placebo: 42.5 ± 13.79, VD3: 41.35 ± 13.58	28/12
Ghazavi et al. [19]	Iran	2023	Nonrandomized trial	60, case: 29, control: 31	Case: 36.03 ± 10.71, control: 29.90 ± 11.99	32/28

Study Outcome Measures

More than half of the studies reported the presence of nasal polyps in CRS, and participants from four studies had CRS without nasal polyps. Sansoni et al. reported a negative correlation between vitamin D levels and CRS. The vitamin D levels in the control group were 19.8 ± 8.9 ng/mL and in the CRS group with nasal polyps were 33.4 ± 11.0 ng/mL [17]. Bavi et al. reported quite low levels of vitamin D in CRS with nasal polyps at 12.11 ± 6.27 ng/mL, while in the control group, the levels were 90 ± 17.18 ng/mL. There was a negative correlation between serum vitamin D3 level and the Lund-Mackay score (R = -0.66) [11]. Similarly, Chandrakar et al. described vitamin D levels of 12.01 ± 7.29 ng/mL in cases, while in controls, the levels were 22.87 ± 14.95 ng/mL. This study also reported a negative correlation with serum levels of 25-hydroxy vitamin D [12]. Hashemian et al. reported vitamin D levels of 17.81 ± 4.46 ng/mL in placebo and 17.61 ± 4.49 ng/mL in vitamin D3 groups [18], while a study by Ghazavi et al. reported vitamin D levels of 19.21 ± 5.45 ng/mL in cases and 18.67 ± 5.13 ng/mL in controls [19]. Zand et al. described a negative correlation with SNOT-22, the Lund-Mackay score, and serum vitamin D levels, with mean vitamin D levels of 24.6 ± 16.9 ng/mL [13]. Likewise, Kalinczak et al. demonstrated that levels of vitamin D in group 1 were 28.02 ng/mL, while in group 2, they were 23.01 ng/mL [15]. Tomaszewska et al. revealed a positive correlation with Lund-Mackay scores (R = -0.42) and a negative correlation with SNOT-22 scores (R = -0.3)m while the levels of vitamin D reported were 28.4 ng/mL in CRS without nasal polyps and 21.0 ng/mL in CRS with nasal polyps, whereas in control groups, the levels were 30.9 ng/mL [16]. Wang et al. [14] noted that vitamin D levels in CRS with nasal polyps were 38.2 ± 9.1 nmol/L, while in CRS without nasal polyps and controls, levels were 48.94 ± 12.1 nmol/L and 54.1 ± 17.1 nmol/L, respectively (Table 4).

**Table 4:** Outcome measures of the included studies. NR: not reported; CRSsNP: chronic rhinosinusitis without nasal polyps; CRSwNP: chronic rhinosinusitis with nasal polyps; LMS: Lund-Mackay score; R: correlation coefficient

Author	Vitamin D levels (mean ± SD)	Prescence of nasal polyps	Correlation
Bavi et al. [11]	CRSwNP: 12.11 ± 6.27 ng/mL vs. control group: 90 ± 17.18 ng/mL	Yes	Negative with serum vitamin D3 level and the Lund-Mackay score (R = -0.66), and Lund-Kennedy score (R = -0.71), and Sino-Nasal Outcome Test-22 (R = -0.49)
Chandrakar et al. [12]	cases 12.01 ± 7.29 ng/mL vs. controls 22.87 ± 14.95 ng/mL	No	Negatively with serum levels of 25-hydroxy vitamin D and positively with hs-CRP (R = 0.658)
Zand et al. [13]	Mean: 24.6 ± 16.9 ng/mL	No	Negatively with SNOT-22 and serum levels of vitamin D, negative LMS, and serum vitamin D levels
Wang et al. [14]	CRSwNP: 38.2 ± 9.1 nmol/L, CRSsNP: 48.94 ± 12.1 nmol/L, control, 54.1 ± 17.1 nmol/L	Yes	NR
Kalińczak-Górna et al. [15]	Group 1: 28.02 ng/mL, group 2: 23.01 ng/mL	No	NR
Tomaszewska et al. [16]	CRSsNP: 28.4 ng/mL, CRSwNP: 21.0 ng/mL, control groups: 30.9 ng/mL	Yes	Positively CT Lund-Mackay scoring (R = -0.42), H scores for VDR (R = 0.28), and negative with SNOT-22 scores (R = -0.3) in the sinus mucosa CRSsNP
Sansoni et al. [17]	Control: 19.8± 8.9 ng/mL, CRSsNP: 33.4 ± 11.0 ng/mL	Yes	Negative with 25-VD3, RANTES (R = -0.612)
Hashemian et al. [18]	Placebo: 17.81 ± 4.46 ng/mL vs. VD3 groups: 17.61± 4.49 ng/mL	No	NR
Ghazavi et al. [19]	Case: 19.21 ± 5.45 ng/mL vs. control: 18.67 ± 5.13 ng/mL	No	NR

Discussion

Various CRS subtypes are caused by diverse inflammatory causes, yet the pathophysiology of CRS is still unknown. Vitamin D3 insufficiency may be related to CRS with nasal polyposis because it has been proven to influence inflammatory mediators in several disorders. According to the findings of several studies, vitamin D3 functions as a steroid hormone with anti-inflammatory properties and is crucial for controlling dendritic cells. The mechanism by which vitamin D3 affects the immune system is comparable functioning of corticosteroids. Numerous studies have demonstrated that vitamin D3 can suppress immune cell differentiation and cytokine production. Some studies indicate that local calcitriol and tacalcitol block the production/synthesis of pro-inflammatory cytokines interleukin (IL) 6 and 8 in fibroblast cultures and that local regulation of vitamin D in the sinonasal tissue during CRS may be independent of serum 25(OH)-D levels [11].

Compared to CRS patients without nasal polyps, vitamin D3 deficiency is more common in CRS patients with nasal polyps, especially in African American communities. Additionally, it has been linked to larger-sized nasal polyps and more severe disease on sinus CT scans. Additionally, in individuals having CRS with nasal polyps and allergic fungal rhinosinusitis, vitamin D3 status correlates adversely with systemic dendritic cell counts and bone degradation. Furthermore, vitamin D3 insufficiency is quite common in pediatric CRS patients with nasal polyps, and almost 90% of pediatric patients with acute fungal rhinosinusitis and CRS with nasal polyps were reported to be vitamin D3 deficient compared to an estimated 18% of children in the general population who are vitamin D3 deficient [20].

The findings of our review demonstrate a negative correlation between levels of vitamin D and CRS. More than half of our included studies reported low levels of vitamin D; additionally, the presence of nasal polyps was observed in five of our included studies. Similar to our findings, Abd Ali et al. reported that when compared to individuals without nasal polyps who had CRS, patients with nasal polyps had considerably lower serum levels of vitamin D, with mean concentrations of 17.9 ± 5.9 ng/mL and 27.3 ± 7.5 ng/mL, respectively (p < 0.001). Vitamin D levels and the severity of CRS also exhibited a negative association (r = -0.431, p = 0.026, odds ratio = 14.3%). Lack of vitamin D greatly increased the likelihood of developing nasal polyps and the severity of CRS [21]. Similarly, the results of a meta-analysis reported that there was a significant correlation between decreased serum vitamin D status and CRS, particularly in patients with CRS with nasal polyps. As a result, serum vitamin D levels may be related to CRS in patients. However, the authors suggested further thorough investigations are necessary for a clearer understanding of this association [22].

Likewise, Wang et al. reported that comparing CRS with nasal polyps to CRS without nasal polyps and control participants, CRS with nasal polyps had lower levels of circulating 25 vitamin D3, and these levels are related to the subjective severity of the disease [14]. Zand et al. reported that in patients with CRS and nasal polyps, there was a strong correlation between serum vitamin D levels and disease severity. Therefore, serum vitamin D levels can be added to the standard workup for individuals with CRS and nasal polyps [13]. On the contrary, the results of a retrospective review did not establish a causal relationship between low vitamin D3 and sinus inflammation, despite the link between vitamin D3 and mucosal thickness on CT scans being demonstrated. Furthermore, the finding that normal vitamin D3 levels were present in 45% of CRS patients with nasal polyps refutes the idea that vitamin D3 is the cause of the condition. The phenotypic CRS with nasal polyps presentation may be influenced by several factors in a specific host, including vitamin D3 deficiency. In some cases, low vitamin D3 is linked to a more severe clinical presentation, as determined by the Lund-Mackay score. The authors additionally recommended that before advising extensive supplementation procedures, a deeper understanding of the function of vitamin D3 in distinct types of CRS with nasal polyps is required [23].

Our findings are, however, in line with the findings of the majority of studies in the literature. As Thakur and Potluri revealed, according to the radiological and endoscopic scoring systems, CRS was moderate to severe, and the CRS group demonstrated a moderately negative relationship between vitamin D and the Lund-Mackay score. Both the CRS with nasal polyps and CRS without nasal polyps subgroups demonstrated a similar pattern. Research in recent years has linked vitamin D deficiency to CRS pathogenesis as one of the key causes. However, there are not many studies available that define the relationship between vitamin D levels and CRS severity [24]. Murdaca et al. showed that in addition to promoting the release of IL-10 and decreasing CD4+ T-cell production of key Th2 cytokines, including IL-4, IL-5, and IL-13, vitamin D can also affect IL-8 expression because 1-hydroxylase has been demonstrated to lower IL-8 gene expression in fibroblasts and keratinocytes in sinonasal tissue. Increased levels of IL-6 and IL-8 may contribute to the pathophysiology of original alterations as well as relapses of chronic sinusitis and nasal polyps, according to recent studies. A statistically significant reduction in vitamin D receptor (VDR) nuclear staining in patients with CRS without nasal polyps and CRS with nasal polyps compared to controls, as well as the existence of VDR protein expression in the sinonasal mucosa. Human sinonasal fibroblasts are VDR-expressing cells thought to be involved in the etiology of CRS with nasal polyps. These play a role in the activation of inflammatory cells, tissue edema, extracellular matrix formation, and tissue remodeling. Furthermore, fibroblasts implicated in the development of nasal polyps might drastically reduce the release of matrix metalloproteinase-2 and matrix metalloproteinase-9 when tumor necrosis factor is present. Low vitamin D levels may encourage greater cytokine production from inflammatory cells and fibroblasts, given the role vitamin D plays in the pathophysiology of CRS and nasal polyps. This might be the cause of nasal polyposis’s severity and the persistence of chronic inflammatory sinus disorders. Various studies have reported an association between serum vitamin D levels and illness severity in people with CRS and nasal polyps [25].

Ebiary et al. reported that patients with CRS and nasal polyps had considerably low vitamin D levels, which suggests a possible causal connection. Without nasal polyps, there was no correlation between vitamin D levels and CRS [26]. However, the results of our study showed that a negative correlation was also observed in CRS without nasal polyps, as reported by the three included studies. Kalinczak et al. narrated that lower serum vitamin D3 levels and a slower healing process were caused by the greater status of the sinus inflammatory process. However, following surgery, the serum levels of vitamin D3 increased in both groups, with the increase being greater in patients with less severe illnesses, as measured by the Lund-MacKay scale. When treatment begins, 25 vitamin D3 is converted to 1.25 vitamin D3, and cathelicidin, an antimicrobial peptide, is produced. The acute-phase reaction is inhibited by vitamin D3, and inflammation resulting from CRS may cause vitamin D3 levels to drop [15].

Ibrahim and Elnimeiri reported a case of a 26-year-old woman who had chronic anosmia and recurrent nasal polyps and presented with a non-classical example of vitamin D deficiency [27]. Her vitamin D insufficiency was identified, and the vitamin D replacement therapy worked effectively for her. This example showed a connection between low vitamin D levels and recurring nasal polyps, which eventually caused chronic anosmia due to persistent high-sensitivity reactions caused by the patient’s immunological system. Asthma and allergic rhinitis are linked in the literature to CRS with nasal polyps, but the cellular and molecular mechanisms behind the clinical symptoms are still poorly understood. Defects in the sinonasal epithelial cell barrier, greater contact with pathogenic and colonized microorganisms, and deregulation of the host immune system are all regarded as important factors in the etiology of illness [27].

Limitations and future research directions

The majority of the studies in our review reported the negative correlation between CRS severity and vitamin D levels; however, due to the scarcity of studies in this regard, more elaborate understanding and studies with evidence-based findings from clinical trials are needed to define any causal relationship between these two entities. As the association of vitamin D deficiency with CRS is still in its evolving phase, the existing studies in the literature are limited in defining any associations, especially in terms of evidence from randomized control trials. Even in our review, only three studies were randomized control trials, and the majority were only observational studies. This necessitates the need for further clinical research to address the association based on strong evidence from various randomized control trials and population-based studies, with specific emphasis on studying vitamin D levels among CRS patients which can aid in developing more targeted and optimal therapeutic strategies in addition to comprehensive understanding of the pathophysiology behind this association. The systematic search methodology and the analysis of all keywords in this field are this study’s main advantages and strengths. Another benefit of this review is that it includes studies from the last 20 years. This time frame allowed the inclusion of the maximum number of studies as the relationship of vitamin D deficiency to CRS is still evolving and under investigation. Furthermore, this study includes findings from randomized trials as well; however, the exclusion of non-English studies which may have resulted in missing some of the potential studies describing significant associations or relationships between vitamin D and CRS and excluding patients with liver and chronic kidney diseases are the limitations of our study. In the future, we can study the association of vitamin D and CRS among different cohorts with different comorbidities.

## Conclusions

CRS and vitamin D levels share a negative/inverse relationship, and quite low levels of vitamin D are reported in CRS patients, making vitamin D an independent prognostic factor for CRS. The degree of CRS, as measured by endoscopic and radiological scores, is moderately inversely correlated with vitamin D intake. Therefore, it is recommended that the relationship between various serum vitamin D concentrations and the severity of CRS be investigated in detail, along with an understanding of the cellular effects of vitamin D levels on the nasal mucosa. Additionally, the therapeutic effects of vitamin D in CRS patients should be backed by evidence-based findings from clinical trials.
